# Effect Range of the Material Constraint-I. Center Crack

**DOI:** 10.3390/ma12010067

**Published:** 2018-12-25

**Authors:** Jie Yang, Lei Wang

**Affiliations:** School of Energy and Power Engineering, University of Shanghai for Science and Technology, Shanghai 200093, China; wl_21th@126.com

**Keywords:** material constraint, effect range, fracture resistance curve, strain field, center crack

## Abstract

Material constraints are important factor effects on the fracture behavior of welded joints. The effect range of the material constraint is an important and interesting issue which needs to be clarified, including whether the effect range of a material constraint exists or not, who will affect it, and whether the material constraint is affected by the no adjacent area or not. In this study, different basic models which reflect different single metallic welded joints, bimetallic welded joints and dissimilar metal welded joints were designed, and the fracture resistance curves and crack tip strain fields of the different models with various material constraints were calculated. Based on the results, the questions above were answered. This study has significance for developing solid mechanics, optimizing joint design, structure integrity assessment, and so on.

## 1. Introduction

Constraint is the resistance of a structure or specimen against plastic deformation [1]. In recent years, the constraint effect due to structure or specimen geometry have been investigated as an important factor affecting the stress distribution around a crack. Some constraint parameters, such as *T* [2], *Q* [3,4], *A_2_* [5], *T_Z_* [6,7,8], have been established to represent the stress fields at the crack tip under different geometry constraint conditions. In addition, the constraint effect due to material strength mismatch, which be called the material constraint, is also an important factor effects on the fracture behavior of material.

The material constraint was firstly demonstrated by Joch et al. [9] and Burstow et al. [10] to show how the slip-line fields were changed by altering the yield strength of the base material. Then, Zhang et al. [11] analyzed a two-material problem where the crack was located in the interface of two dissimilar materials and established a material constraint parameter *M* to consider the effect of strength mismatch on crack tip stress fields, as follows:

(1) $$M=\frac{\sigma_{Yw}}{\sigma_{Yb}},$$

where the $\sigma_{Yw}$ is the yield stress of the weld material and $\sigma_{Yb}$ is the yield stress of the base material. They also proposed that the stress fields of an interface crack in a mismatched problem could be obtained using the *J-Q-M* formulation, which was derived by extending the *J-Q* theory. Betegón et al. [12] defined a procedure similar to the *J-T*, also by establishing an additional parameter $\beta_{m}$ that quantifies the material constraint, and a total constraint parameter $\beta_{T}$ was defined as follows:

(2) $$\beta_{T}=\beta_{m}\cdot \sqrt{\frac{a}{h}}+\beta_{g},$$

where $\beta_{m}$ is a constraint parameter defined for the overmatched welded joints to quantify the material constraint effect on the crack tip stress fields, $\beta_{g}$ is a geometry parameter by means of the *T*-stress to quantify the geometry constraint, *a* is crack length and *h* is weld semi-width. Recently, the author [13,14,15] defined a unified constraint parameter $A_{p}$ based on the areas surrounded by the equivalent plastic strain ($\varepsilon_{p}$) isolines ahead of the crack tip to characterize both geometry and material constraint. The unified constraint parameter $A_{p}$ was defined as follows:

(3) $$A_{p}=\frac{A_{PEEQ}}{A_{ref}},$$

where $A_{PEEQ}$ is the areas surrounded by the $\varepsilon_{p}$ isolines ahead of the crack tip and $A_{ref}$ is the reference areas surrounded by the $\varepsilon_{p}$ isolines in a standard test.

Furthermore, many scholars focused their studies on the fracture behavior of bi-material affected by the material constraint. Negre et al. [16] and Samal et al. [17] investigated the altering of fracture resistance and crack path deviation in the bi-material interface region affected by the material constraint. Fan et al. [18,19,20] studied the *J*-resistance curves, fracture toughness, crack growth paths and stress triaxiality of bi-materials under different work-hardening mismatches. Besides, some scholars focused their studies on the fracture behavior of dissimilar metal-welded joints affected by the material constraint. Rakin et al. [21] investigated the fracture behaviors of the over-matched and under-matched high-strength low-alloyed steel weld joints. Wang et al. [22,23] studied the local fracture resistances and crack growth paths of a dissimilar metal-welded joint at different crack positions with different material constraints. Xue et al. [24] investigated the stress and strain of a micro region influenced by material yield strength mismatch at the crack tip of a dissimilar metal-welded joint. Zhu et al. [25] studied the stress fields of a crack tip affected by material constraints in a nuclear pressure steel A508-III dissimilar metal welded joint.

These studies clarified the effect of a material constraint on the fracture behaviors of welded joints, and laid the foundation for the building of accurate structure integrity assessment. Nevertheless, most studies focus their attention on the strength mismatch of both sides of the crack, such as over-match, under-match, and so on. There is another interesting and important issue, the effect range of the material constraint, which also needs to be clarified. This includes whether exists the effect zone or not, who effects it, whether the material constraint affected by the no adjacent area or not, and so on. Solving this issue is of significance in developing solid mechanics, optimizing joint design and structure integrity assessment. 

Thus, in this study, different basic models which represent different single metallic-welded joints, bimetallic-welded joints and dissimilar metal-welded joints were designed. Then, the fracture resistance curves and crack tip strain fields of different models under different material constraints were calculated. Based on the results, the questions above were answered, and the effect range of the material constraint was investigated.

## 2. Materials and Models Design

### 2.1. Materials

**Figure 1:**
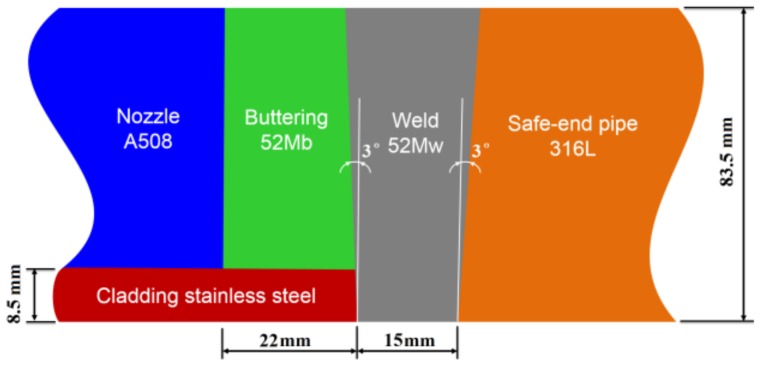
A dissimilar metal welded joint in a nuclear power plant.

Four different materials (A508, 52Mb, 52Mw and 316L) selected from a dissimilar metal-welded joint (DMWJ) in nuclear power plant were used in this study, as shown in Figure 1. The DMWJ was fabricated by Shanghai Company of Nuclear Power Equipment (Shanghai, China). The DMWJ was a full scale mock up of the DMWJ in a nuclear power plants. During the manufacturing process, the base metal A508 was pre-heated to 125 °C before buttering to prevent weld cracking. The buttering layer was deposited using a 1.2 mm diameter Alloy52M welding wire using automatic gas-tungsten arc welding (GTAW) on the ferritic nozzle face. The welding current, voltage and speed were 200 A, 11.5 V and 1.85 mm/s, respectively. A total of 478 weld passes were deposited, and the buttering layer with an average width of 20 mm was formed. Then, heat treatment (annealing at 610 °C for 15 h, with subsequent furnace cooling to 300 °C) was conducted on the buttering to relieve the residual stress. Thereafter, 100% non-destructive testing was performed on the buttering. This buttering layer material is denoted as buttering 52Mb.

After buttering, welding was carried out between the buttering layer and the austenitic safe-end pipe using GTAW and a 0.9 mm diameter Alloy52M welding wire. The welding current, voltage and speed were 180 A, 10 V and 1.75 mm/s, respectively. A total of 439 weld passes were deposited, and the weld with an average width of 19 mm was formed. After welding, 100% nondestructive testing was performed again on the weld. This weld metal material is denoted as weld 52Mw.

**Figure 2:**
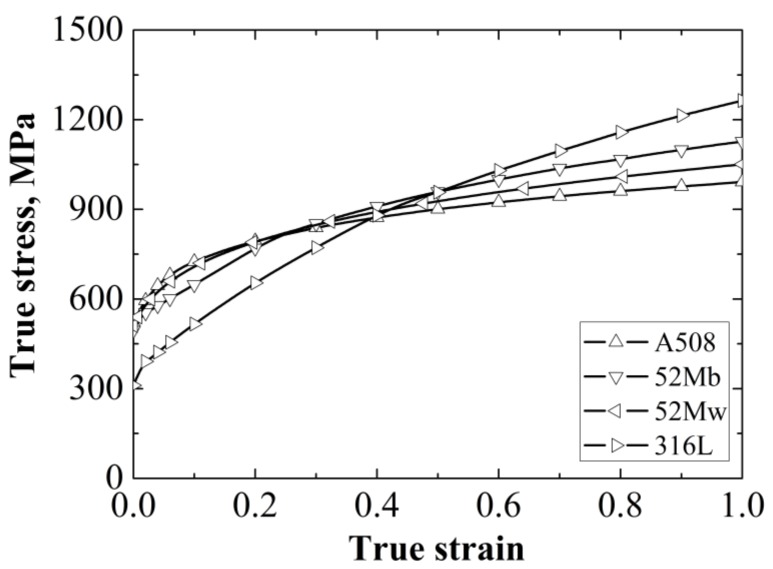
The true stress-strain curves of four materials. Reprinted from Materials Science and Engineering A, 568, Wang, H.T.; Wang, G.Z.; Xuan, F.Z.; Liu, C.J.; Tu, S.T.; Local mechanical properties of a dissimilar metal welded joint in nuclear power systems, 108, Copyright 2013, with permission from Elsevier. [22].

The true stress-strain curves of the four different materials at room temperature have been obtained [22], as shown in Figure 2. The measured Young’s modulus *E* of A508, 52Mb, 52Mw and 316L are 202,410 MPa, 178,130 MPa, 178,130 MPa and 156,150 MPa, respectively, and the Poisson’s ratios *ν* of them are 0.3 [22].

### 2.2. Models Design

**Figure 3:**
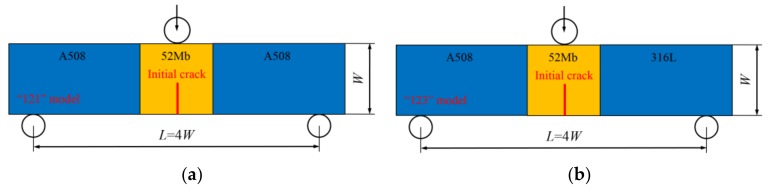

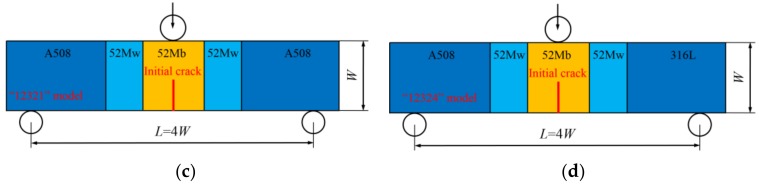
Four different basic models, (**a**) “121” model, (**b**) “123” model, (**c**) “12321” model and (**d**) “12324” model.

Single-edge-notched bend (SENB) specimens were used in this study, and four different basic models were designed, which contain the “121” model, “123” model, “12321” model and “12324” model, as shown in Figure 3.

These models are similar to sandwiches. The “121” model means the model contains “1” and “2” two kinds of materials, the materials in the model from left to right are “1”, “2” and “1”. The “123” model means the model contains “1”, “2” and “3” three kinds of materials, the materials in the model from left to right are “1”, “2” and “3”. The “12321” model means the model contains “1”, “2” and “3” three kinds of materials, the materials in the model from left to right are “1”, “2”, “3”, “2” and “1”. The “12324” model means the model contains “1”, “2”, “3” and “4” four kinds of materials, the materials in the model from left to right are “1”, “2”, “3”, “2” and “4”. Furthermore, the “121” model represents the single metallic welded joint, the “123” model represents the bimetallic welded joint, the “12321” and “12324” models represent the dissimilar metal welded joint.

For all the models, a load roll is applied at the top and center of the SENB specimen, and two back-up rolls are applied at the bottom of the SENB specimen. The loading is applied at the load roll by prescribing a displacement of 6 mm, and the two back-up rolls are fixed by control displacement and rotation. The initial crack is located in the middle of specimen. All the specimen widths are 14.4 mm (*W* = 14.4 mm), the loading spans are 57.6 mm (*L* = 4*W*), the specimen thicknesses *B* are 12 mm, and the initial crack lengths are 7.2 mm (*a/W* = 0.5). 

Different material constraints were obtained by changing the width (the direction perpendicular to the initial crack) of 52Mb or 52Mw in each model. For the “121” and “123” models, changing the width of 52Mb from 0 to 80 mm. When the width of 52Mb is 0 mm, the welded joint is the same as the A508 and 316L homogeneous material models, respectively; when the width of 52Mb is 80 mm, the welded joint is same as the 52Mb homogeneous material model. For the “12321” and “12324” models, the widths change to 52Mb and 52Mw, respectively. When changing the width of 52Mb individually, the width of 52Mw is fixed to 1mm, and changing the width of 52Mb from 0 to 80 mm; when changing the width of 52Mw individually, the width of 52Mb is fixed to 1 mm, and changing the width of 52Mw from 0–39.5 mm.

### 2.3. Gurson-Tvergaard-Needleman (GTN) Damage Model

Ductile crack growth in metals is a result of nucleation, growth and coalescence of micro voids. In order to obtain the fracture resistance curves of different models, the finite element method (FEM) simulation based on Gurson-Tvergaard-Needleman (GTN) damage model was used in this study. There are nine parameters in the GTN damage model: the constitutive parameters *q_1_*, *q_2_* and *q_3_*, the void nucleation parameters *ε_Ν_*, *S_N_* and *f_N_*, the initial void volume fraction *f_0_*, the critical void volume fraction *f_C_* and the final failure parameter *f_F_*. The void coalescence occurs when the void volume fraction reaches the critical value *f_C_*, and the fracture occurs when the void volume fraction reaches the final value *f_F_*. These parameters have been obtained and listed in Table 1 [26]. 

**Figure 4:**
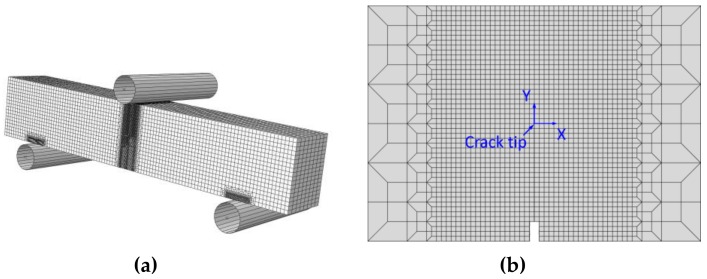
The whole mesh of the typical model (**a**) and the mesh in the crack growth region (**b**).

This GTN damage model has been implemented in the ABAQUS code (6.14, Dassault Systèmes group company, Shanghai, China), and is widely used to simulate the crack propagation process and calculate the *J*-resistance curve. During the finite element analysis, the 3D eight-node isoperimetric element with reduced integration (C3D8R) are used [13,14]. The typical finite element mesh for the “121” model with *W*_52Mb_ = 16 mm is illustrated in Figure 4a, the minimum size of mesh in the crack growth region is 0.1 mm × 0.1 mm [27], as shown in Figure 4b. This typical model contains 75,872 elements and 87,849 nodes. In addition, the surface-to-surface contact (explicit) interaction type was used in the model. Moreover, the sliding formulation is finite sliding, the mechanical constraint formulation is kinematic contact method.

The load versus load-line displacement curve can be obtained from the FEM simulation. With instantaneous crack lengths obtained at each loading point, a crack growth resistance curve can be determined, as specified in ASTM (American Society for Testing and Materials) E1820 [28].

## 3. Results and Discussion

### 3.1. “121” Model

**Figure 5:**
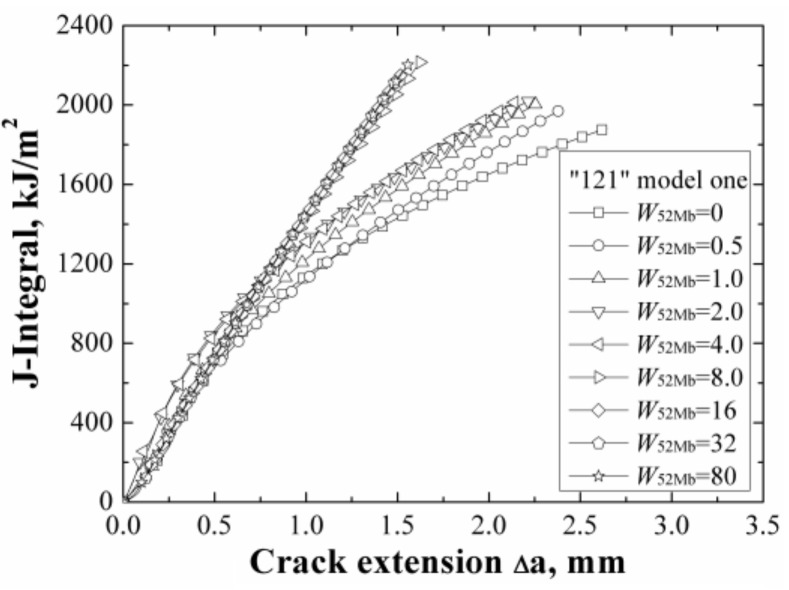
The *J*-resistance curves of different “121” models.

The *J*-resistance curves of different “121” models under different material constraints are shown in Figure 5. It can be found that increasing of the width of 52Mb from 0 to 8 mm, the *J*-resistance curves of the “121” models increase. When the width of 52Mb is up to 8 mm, the *J*-resistance curves remain steady and will not change with the increasing of the 52Mb’s width. 

Because material A508 has lower strength than material 52Mb, the “121” model is an over-matched joint. In this model, when the *W*_52Mb_ = 0 mm, it is the same with the homogeneous material A508; when the *W*_52Mb_ = 80 mm, it is the same with the homogeneous material 52Mb. Thus, the results in Figure 5 show that for an over-matched joint, the *J*-resistance curve of the joint is higher than the base material. In addition, a notable phenomenon is that when the width of 52Mb is up to 8 mm, the *J*-resistance curves of the “121” models are same with the *J*-resistance curve of homogeneous material 52Mb. It means that the crack is out of the effect range of the material constraint induced by the A508/52Mb interface. In this condition, it does not matter even if the material on the outside is soft or hard. That is, when the crack locates out of the effect range of material constraint, the fracture resistance curve of the weld joint no longer influenced by the material constraint anymore. Of course, the effect range is also related to different materials and models.

**Figure 6:**
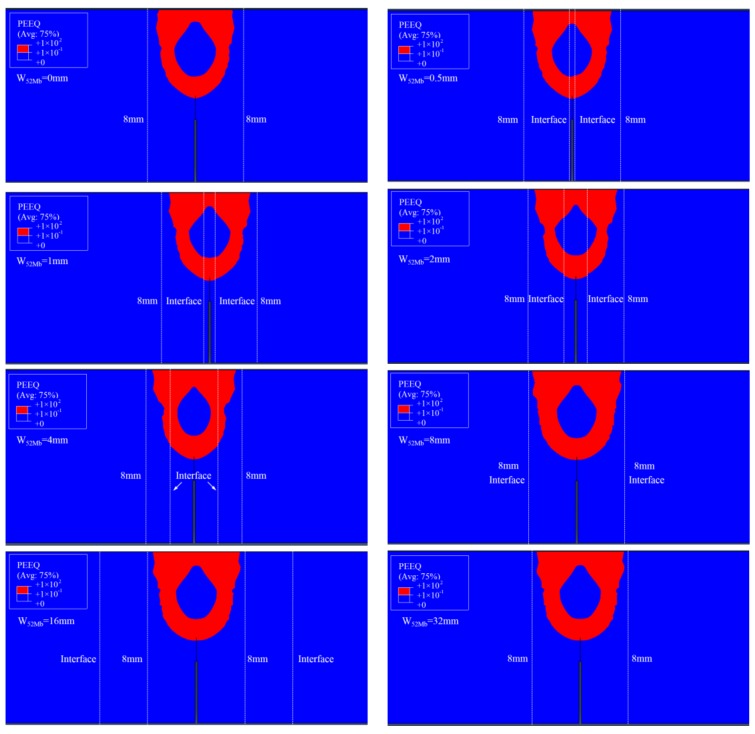

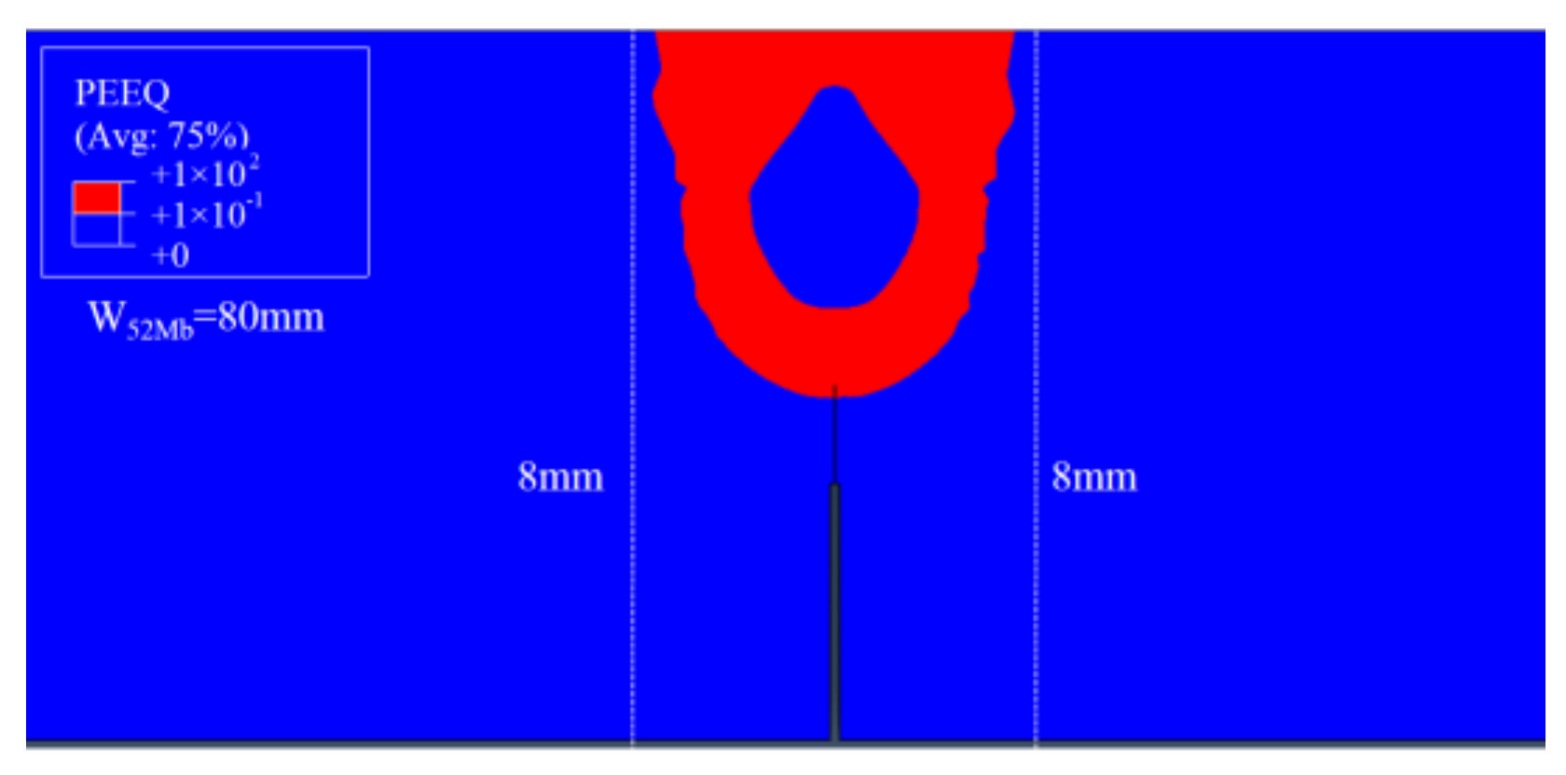
The distributions of *ε_p_* = 0.1 isoline at crack tip at *J* = 1600 kJ/m^2^.

Figure 6 shows the distributions of equivalent plastic strain *ε_p_* = 0.1 isoline at crack tip at the same *J*-integral (*J* = 1600 kJ/m^2^) for different “121” models. It can be found that though the distributions of equivalent plastic strain are different for different models, but the equivalent plastic strains surrounded by *ε_p_* = 0.1 isoline are within the scope of 8 mm for all the models. When the interface is located within this scope, the *J*-resistance curve will be affected by the material constraint; when the interface is located outside this scope, the *J*-resistance curve will not be affected by material constraint. This scope is the effect range of the material constraint.

**Figure 7:**
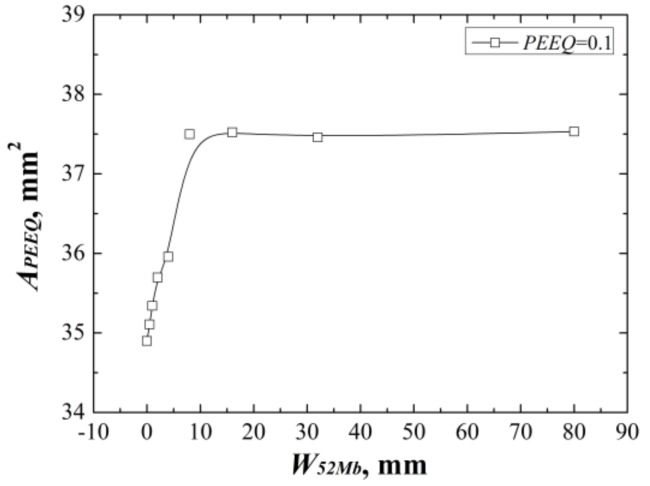
The areas surround by the *ε_p_* = 0.1 isoline at the same *J*-integral for “121” model.

In addition, the areas surround by the *ε_p_* = 0.1 isoline reflect the same change rule with the *J*-resistance curves, as shown in Figure 7. Because the constraint is the resistance of a structure against plastic deformation, at the same *J*-integral (driving force) a lower plastic deformation reflects a higher constraint and a lower *J*-resistance curve, and vice versa. The same change rules between *J*-resistance curves and areas can prove each other and also reflect the change rules are related to the constraint. 

Furthermore, Figure 7 also shows that the *J*-resistance curve of material is controlled by the strain fields at crack tip rather than stress fields. It should be noted that the *ε_p_* = 0.1 isoline was selected here, when a small *ε_p_* value was selected, the scope will beyond the 8 mm. Therefore, there may exist a main control value or control zone. For this study, the main control value is *ε_p_* = 0.1.

### 3.2. “123” Model

**Figure 8:**
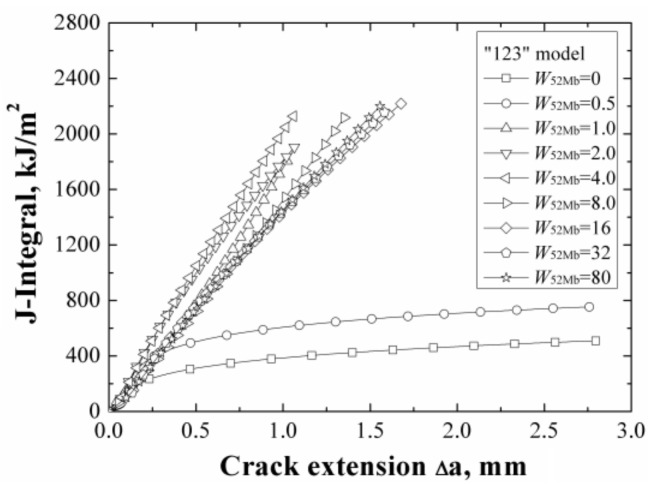
The *J*-resistance curves of different “123” models.

The *J*-resistance curves of different “123” models under different material constraints are shown in Figure 8. It can be found that increasing of the width of 52Mb, the *J*-resistance curves of the models increase firstly then decrease, and finally remain steady. The model with *W*_52Mb_ = 0 mm has the lowest *J*-resistance curve and the model with *W*_52Mb_ = 4 mm has the highest *J*-resistance curve. When the width of 52Mb is up to 16 mm, the *J*-resistance curve will not change with increasing of the 52Mb’s width.

When the *W*_52Mb_ = 0 mm, the model is the same with the bimetallic welded joint with an interface crack. In this condition, the model has the lowest *J*-resistance curve, which shows that the interface crack in bimetallic welded joint is very dangerous. With increasing of the width of 52Mb, the *J*-resistance curve of the model increases. When the *W*_52Mb_ = 4 mm, there exists an optimal width and the model has the highest *J*-resistance curve. Then, the *J*-resistance curves of the models decrease and remain steady at last.

The same with the “121” model, when the width of 52Mb up to a value, the *J*-resistance curve of the model is same with the *J*-resistance curve of homogeneous material 52Mb. That is, an effect range also exists. By contrast with the “121” model, the steady value is different and is related to the materials on both sides of the crack.

**Figure 9:**
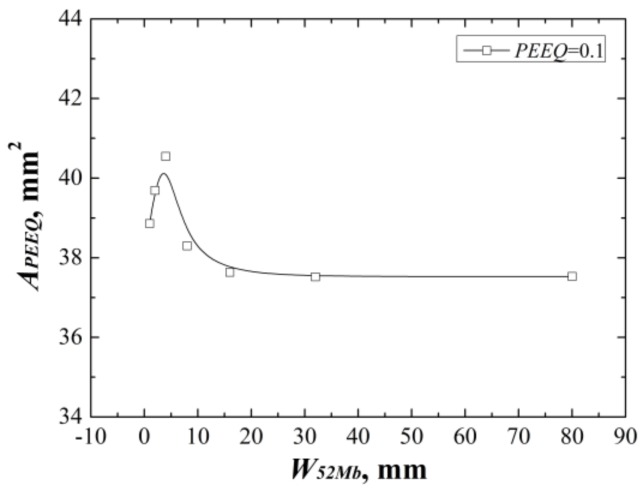
The areas surround by the *ε_p_* = 0.1 isoline at the same *J*-integral for “123” model.

Figure 9 shows the areas surround by the *ε_p_* = 0.1 isoline at crack tip at the same *J*-integral (*J* = 1600 kJ/m^2^) for different “123” models. It reflects the same change rule with the *J*-resistance curves. The same change rules can prove each other also.

### 3.3. “12321” Model

**Figure 10:**
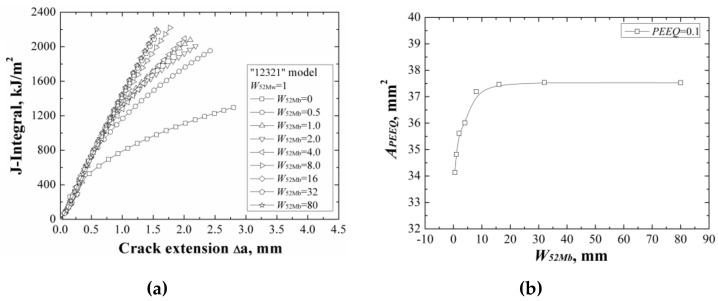
The *J*-resistance curves (**a**) and the areas surround by the *ε_p_* = 0.1 isoline (**b**) of different “12321” models with the same *W*_52Mw_.

When changing the width of 52Mb individually, the *J*-resistance curves of different “12321” models under different material constraints are shown in Figure 10a. It can be found that increasing the width of 52Mb, the *J*-resistance curves of different “12321” models increase and remain steady when the width of 52Mb is up to 16 mm. It is similar with the “121” model; the strength of the material 52Mb is higher than the materials A508 and 52Mw, with increasing of the width of 52Mb, the *J*-resistance curve of the model increases. When the width of 52Mb over the effect range of the material constraint, the *J*-resistance curve of the “12321” model is unchanged.

The areas surrounded by the *ε_p_* = 0.1 isoline at crack tip at the same *J*-integral (*J* = 1600 kJ/m^2^) for different “12321” models are shown in Figure 10b, which also reflects the same change rule with the *J*-resistance curves.

**Figure 11:**
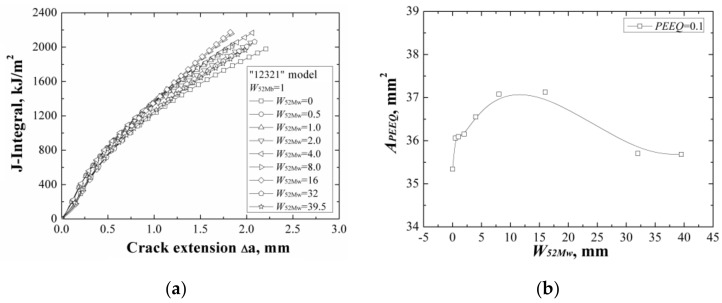
The *J*-resistance curves (**a**) and the areas surround by the *ε_p_* = 0.1 isoline (**b**) of different “12321” models with the same *W*_52Mb_.

When changing the width of 52Mw individually, the *J*-resistance curves of different “12321” models under different material constraints are shown in Figure 11a. It can be found that increasing of the width of 52Mw, the *J*-resistance curves of different “12321” models increase firstly then decrease, and finally remain steady. The model with *W*_52Mw_ = 0 mm has the lowest *J*-resistance curve and the model with *W*_52Mw_ = 16 mm has the highest *J*-resistance curve. When the width of 52Mw up to 32 mm, the *J*-resistance curve of the model will not change by increasing the 52Mw’s width. 

Because the strength of the material 52Mw is higher than the material A508, thus, increasing of the width of 52Mw, the *J*-resistance curve of the model increases firstly. When the *W*_52Mw_ = 16 mm, there exists an optimal width and the model has the highest *J*-resistance curve. Then, the *J*-resistance curves of the models decrease and remain steady at last when the total width of 52Mb and 52Mw over the effect range of the material constraint.

The areas surrounded by the *ε_p_* = 0.1 isoline at crack tip at the same *J*-integral (*J* = 1600 kJ/m^2^) for different “12321” models are shown in Figure 11b, which also reflects the same change rule with the *J*-resistance curves.

### 3.4. “12324” Model

**Figure 12:**
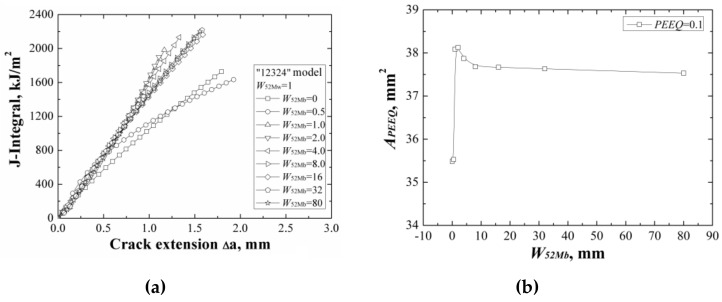
The *J*-resistance curves (**a**) and the areas surround by the *ε_p_* = 0.1 isoline (**b**) of different “12324” models with the same *W*_52Mw_.

When changing the width of 52Mb individually, the *J*-resistance curves of different “12324” models under different material constraints are shown in Figure 12a. It can be found that increasing the width of 52Mb, the *J*-resistance curves of the models increase firstly then decrease, and finally remain steady. The models with *W*_52Mb_ = 0 mm and *W*_52Mb_ = 0.5 mm have the lowest *J*-resistance curves and the model with *W*_52Mb_ = 2 mm has the highest *J*-resistance curve. When the width of 52Mb up to 8 mm, the *J*-resistance curve will not change by increasing the 52Mb’s width.

It is the same with “12321” model, because the strength of material 52Mb is higher than the material 52Mw, and increasing the width of 52Mb, the *J*-resistance curves of the models increase firstly. However, the strength of material 52Mb is lower than the material 316L, the *J*-resistance curves of the models do not always increase. When the *W*_52Mb_ = 2 mm, there exists an optimal width and the model has the highest *J*-resistance curve. Then, the *J*-resistance curves of the models decrease and remain steady at last when the width of 52Mb over the effect range of the material constraint.

The areas surrounded by the *ε_p_* = 0.1 isoline at crack tip at the same *J*-integral (*J* = 1600 kJ/m^2^) for different “12324” models are shown in Figure 12b, which also reflects the same change rule with the *J*-resistance curves.

**Figure 13:**
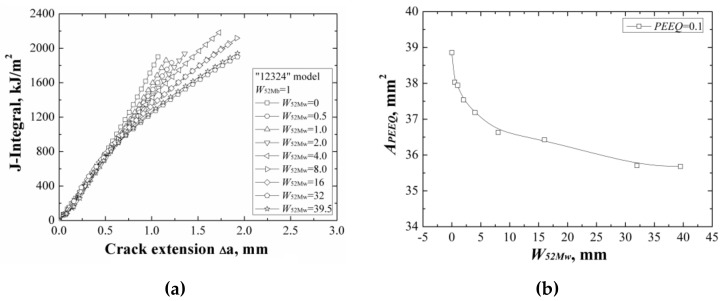
The *J*-resistance curves (**a**) and the areas surround by the *ε_p_* = 0.1 isoline (**b**) of different “12324” models with the same *W*_52Mb_.

When changing the width of 52Mw individually, the *J*-resistance curves of different “12324” models under different material constraints are shown in Figure 13a. It can be found that increasing of the width of 52Mw, the *J*-resistance curves of the “12324” models decrease. When the width of 52Mw is up to 32 mm, the *J*-resistance curves of the models remain steady and will not change with increasing the 52Mw’s width. This is because although the strength of material 52Mw is higher than the material A508, it is much lower than the materials 316L and 52Mb. Increasing of the width of 52Mw, the *J*-resistance curves of the models decrease until the total width of 52Mb and 52Mw over the effect range of the material constraint.

The areas surrounded by the *ε_p_* = 0.1 isoline at crack tip at the same *J*-integral (*J* = 1600 kJ/m^2^) for different “12324” models are shown in Figure 13b, which also reflects the same change rule with the *J*-resistance curves.

In general, the above results show the effect range of the material constraint. Comparing all the models, it can be found that the effect range of the material constraint is real. The effect range relates to the materials on both sides of the crack. It should be pointed that the effect range is not only related to the adjacent material, but also non-adjacent material. It can be proved by comparing the *J*-resistance curves of the “12321” model and “12324” model. When the two cracks have the same adjacent material and dimensions but different non-adjacent materials, the *J*-resistance curves of the two models are different. That is, the *J*-resistance curves influenced by all the materials within the effect range, no matter whether they are adjacent or not.

## 4. Conclusions

In this study, the center crack was selected, different models that reflect different welded joints with various material constraints were designed, and the effect range of the material constraint was studied. The main results obtained are summarized as follows:

(1) For all the weld joints, the effect ranges of the material constraints are real. When the crack locates without the effect range of material constraint, the fracture resistance curves of the weld joints are no longer influenced by the material constraint any more.
(2) The *J*-resistance curves of the weld joints are influenced by all the materials within the effect range, no matter whether the material is adjacent to the crack or not.
(3) The areas surrounded by the *ε_p_* isoline reflect the same change rule with the *J*-resistance curves. The *J*-resistance curves of the materials are controlled by the strain fields rather than the stress fields, and there may exist a main control value or control zone.

## Figures and Tables

**Table 1:** The GTN parameters of different materials. Reprinted by permission from Springer, Copyright 2017. [26]

Material	A508	52Mb	52Mw	316L
*q_1_*	1.5	1.5	1.5	1.5
*q_2_*	1	variable	1	variable
*q_3_*	2.25	2.25	2.25	2.25
*ε_Ν_*	0.3	0.3	0.3	0.3
*S_N_*	0.1	0.1	0.1	0.1
*f_N_*	0.002	0.002	0.002	0.002
*f_0_*	0.00008	0.000001	0.00015	0.000001
*f_C_*	0.04	0.04	0.04	0.04
*f_F_*	0.25	0.25	0.25	0.25

## References

[1] Brocks W., Schmitt W. (1995). The second parameter in J-R curves: Constraint or triaxiality. ASTM Int..

[2] Larsson S.G., Carlsson A.J. (1973). Influence of non-singular stress terms and specimen geometry on small-scale yielding at crack tips in elastic-plastic materials. J. Mech. Phys. Solids.

[3] O’Dowd N.P., Shih C.F. (1991). Family of crack-tip fields characterized by a triaxiality parameter. I. Structure of fields. J. Mech. Phys. Solids.

[4] O’Dowd N.P., Shih C.F. (1992). Family of crack-tip fields characterized by a triaxiality parameter. II. Fracture applications. J. Mech. Phys. Solids.

[5] Chao Y.J., Yang S., Sutton M.A. (1994). On the fracture of solids characterized by one or two parameters: Theory and practice. J. Mech. Phys. Solids.

[6] Guo W. (1993). Elastoplastic three dimensional crack border field-I. Singular structure of the field. Eng. Fract. Mech..

[7] Guo W. (1993). Elastoplastic three dimensional crack border field-II. Asymptotic solution for the field. Eng. Fract. Mech..

[8] Guo W. (1995). Elasto-plastic three-dimensional crack border field-III. Fracture parameters. Eng. Fract. Mech..

[9] Joch J., Ainsworth R.A., Hyde T.H. (1993). Limit load and J-estimates for idealised problems of deeply cracked welded joints in plane-strain bending and tension. Fatigue Fract. Eng. Mater. Struct..

[10] Burstow M.C., Ainsworth R.A. (1995). Comparison of analytical, numerical and experimental solutions to problems of deeply cracked welded joints in bending. Fatigue Fract. Eng. Mater. Struct..

[11] Zhang Z.L., Hauge M., Thaulow C. (1996). Two-parameter characterization of the near-tip stress fields for a bi-material elastic-plastic interface crack. Int. J. Fract..

[12] Betegón C., Peñuelas I. (2006). A constraint based parameter for quantifying the crack tip stress fields in welded joints. Eng. Fract. Mech..

[13] Yang J., Wang G.Z., Xuan F.Z., Tu S.T. (2013). Unified characterisation of in-plane and out-of-plane constraint based on crack-tip equivalent plastic strain. Fatigue Fract. Eng. Mater. Struct..

[14] Yang J., Wang G.Z., Xuan F.Z., Tu S.T. (2014). Unified correlation of in-plane and out-of-plane constraints with fracture toughness. Fatigue Fract. Eng. Mater. Struct..

[15] Yang J., Wang G.Z., Xuan F.Z., Tu S.T. (2014). Unified correlation of in-plane and out-of-plane constraint with fracture resistance of a dissimilar metal welded joint. Eng. Fract. Mech..

[16] Negre P., Steglich D., Brocks W. (2005). Crack extension at an interface: prediction of fracture toughness and simulation of crack path deviation. Int. J. Fract..

[17] Samal M.K., Balani K., Seidenfuss M., Roos E. (2009). An experimental and numerical investigation of fracture resistance behaviour of a dissimilar metal welded joint. Proc. Inst. Mech. Eng. C J. Mech. Eng. Sci..

[18] Fan K., Wang G.Z., Tu S.T., Xuan F.Z. (2016). Geometry and material constraint effects on fracture resistance behavior of bi-material interfaces. Int. J. Fract..

[19] Fan K., Wang G.Z., Xuan F.Z., Tu S.T. (2015). Effects of work hardening mismatch on fracture resistance behavior of bi-material interface regions. Mater. Des..

[20] Fan K., Wang G.Z., Xuan F.Z., Tu S.T. (2015). Local fracture resistance behavior of interface regions in a dissimilar metal welded joint. Eng. Fract. Mech..

[21] Rakin M., Medjo B., Gubeljak N., Sedmak A. (2013). Micromechanical assessment of mismatch effects on fracture of high-strength low alloyed steel welded joints. Eng. Fract. Mech..

[22] Wang H.T., Wang G.Z., Xuan F.Z., Liu C.J., Tu S.T. (2013). Local mechanical properties of a dissimilar metal welded joint in nuclear power systems. Mater. Sci. Eng. A.

[23] Wang H.T., Wang G.Z., Xuan F.Z., Tu S.T. (2013). An experimental investigation of local fracture resistance and crack growth paths in a dissimilar metal welded joint. Mater. Des..

[24] Xue H., Sun J. (2016). Study on micro region of crack tip of welded joints under different matches of yield stress. Hot Work. Technol..

[25] Zhu Z.Q., Jing H.Y., Ge J.G., Chen L.G. (2005). Effects of strength mis-matching on the fracture behavior of nuclear pressure steel A508-III welded joint. Materi. Sci. Eng. A.

[26] Yang J. (2017). Micromechanical analysis of in-plane constraint effect on local fracture behavior of cracks in the weakest locations of dissimilar metal welded joint. Acta Metall. Sin..

[27] Østby E., Thaulow C., Zhang Z.L. (2007). Numerical simulations of specimen size and mismatch effects in ductile crack growth-Part I: Tearing resistance and crack growth paths. Eng. Fract. Mech..

[28] (2008). Standard Test Method for Measurement of Fracture Toughness.

